# A Rare Case of an Abdominal Pedunculated Bronchogenic Cyst

**DOI:** 10.7759/cureus.102874

**Published:** 2026-02-03

**Authors:** Caroline Degreve, Matthias Van Aerde, Livia Dumitru, Philippe Colonval, François Lienard

**Affiliations:** 1 General Surgery, Hôpital Civil Marie-Curie (CHU), Charleroi, BEL; 2 General Surgery, Catholic University of Leuven, Leuven, BEL; 3 Thoraco-Vascular Surgery, CHR De La Haute Senne-Le Tilleriau, Soignies, BEL; 4 Abdominal Surgery, CHR De La Haute Senne-Le Tilleriau, Soignies, BEL

**Keywords:** abdomen, bronchogenic cyst, ectopic cyst, foregut malformation, laparoscopic surgery, minimal invasive surgery

## Abstract

Bronchogenic cysts (BC) are rare congenital malformations that result from abnormal budding of the primitive foregut. Most of these cysts are located in the mediastinum or the lungs. Rarely, abdominal BCs result from embryonic budding migrating below the diaphragm prior to the fusion of the pleuroperitoneal membranes. We present a case of a 34-year-old male patient undergoing evaluation for bronchopneumopathy. Preoperative imaging revealed a well-circumscribed cystic lesion adjacent to the colon, which failed to provide a definitive diagnosis due to its atypical location and nonspecific radiological features. The patient underwent laparoscopic exploration. During the intervention, we discovered a BC connected to the oesophagus. Histopathological analysis confirmed the diagnosis of a BC, demonstrating ciliated pseudostratified epithelium and bronchial wall elements. Follow-up examinations confirmed an unremarkable post-operative course and total recovery. BC are most commonly identified during early childhood and are typically located in the mediastinum or lungs. They should also be considered in the differential diagnosis of incidentally discovered abdominal cystic lesions. There is no consensus on the standard of care; however, surgery provides favorable clinical results and a low risk of morbidity. Our case illustrates that laparoscopic removal is a safe and feasible option when malignancy has been carefully excluded.

## Introduction

Bronchogenic cysts (BC) are rare congenital anomalies accounting for roughly 50% of mediastinal cysts [1]. The condition was initially described in the mid-19th century (1859) [2]. During the migration of the primitive foregut, the cyst can appear anywhere on its path. Thus, the malformation’s localisation depends on the timing of the budding during gestation. Most of them seem to be in the mediastinum and the lungs. Rarely, a BC can appear in the abdominal or retroperitoneal region [3]. The clinical presentation is highly variable. Most BCs are discovered in the first years of life, but sometimes they can be asymptomatic for a long time [2]. When these cysts enlarge or become infected, they may give rise to multiple symptoms, including cough, dyspnea, chest pain, or recurrent respiratory infections [2]. Their rarity and nonspecific imaging in ectopic locations frequently lead to diagnostic uncertainty and misclassification. Once malignancy is excluded, surgical resection is the treatment of choice. This offers excellent postoperative outcomes and reduces patient morbidity.

This article presents a case of a 34-year-old male patient with an abdominal bronchogenic cyst, discusses the diagnostic challenges associated with this uncommon presentation, and reviews current considerations in management and surgical treatment. Furthermore, in our case, intraoperative findings revealed a connection between the cyst and the oesophagus. This observation highlights that bronchogenic cysts can occur in atypical locations and should be considered in the differential diagnosis of intra-abdominal masses.

This article was previously presented as a poster at the Belgian Surgical Week, on April 24, 2025.

## Case presentation

**Figure 1:**
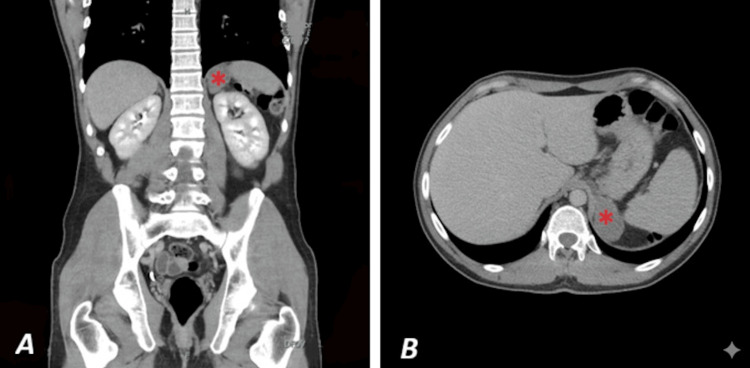
Thoracic contrast-enhanced computed tomography (CT) scan A multilobulated mass-like process with a few small calcifications was observed in the left adrenal compartment, measuring 48 × 31 mm in the axial plane, appearing to extend toward the esophagogastric junction. This lesion demonstrated mild enhancement after intravenous contrast administration, increasing from 24 HU to 42 HU on the portal venous phase. The fat plane was focally disrupted between the mass and the stomach on the one hand, and between the mass and the left adrenal gland on the other. However, this mass did not primarily appear to be of adrenal origin. A. Bronchogenic cyst (*) on CT-scan in coronal plane B. Bronchogenic cyst (*) on CT-scan in axial plane

A 34-year-old male was admitted to the outpatient clinic with bronchopneumopathy. For several weeks, the patient presented with a cough associated with frequent non-infected expectoration. As the cough did not respond to standard treatment, he was referred to and subsequently evaluated by a pulmonary specialist. A thoracic CT scan with contrast showed a 15-mm peripherally hypoenhancing splenic lesion, suggestive of a cyst, in close proximity to the left adrenal gland (Figure 1).

**Figure 2:**
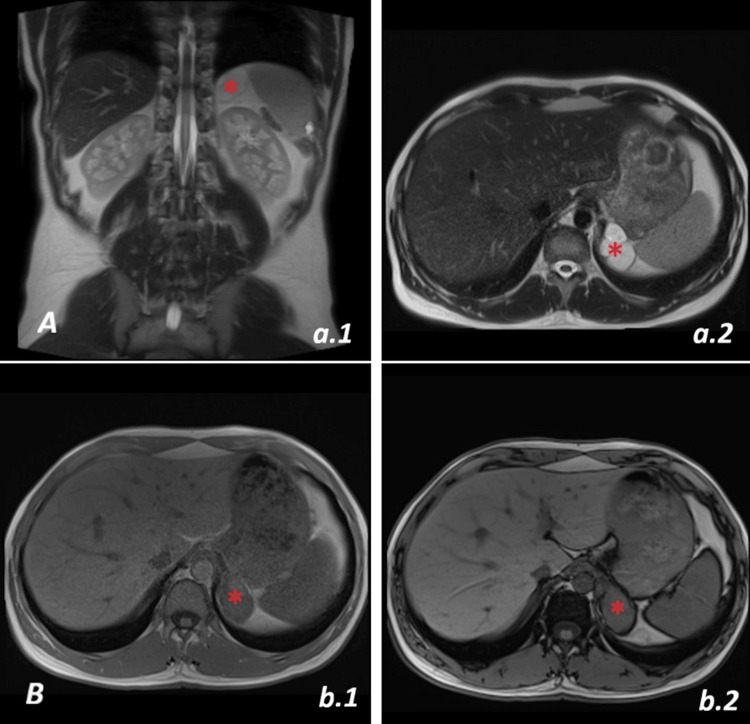
Thoracic magnetic resonance Imagering (MRI) A lesion showing high signal intensity on T2-weighted images and low signal intensity on T1-weighted images, without a fatty component was observed in the left adrenal compartment. A. Bronchogenic cyst (*) on MRI with high intensity on T2 weighted images a.1: In coronal plane; a.2: In axial plane B. Bronchogenic cyst (*) on MRI with low intensity on T1 weighted images (axial view) b.1: In-phase T1-weighted MRI image; b.2: Out-of-phase T1-weighted MR image

The cyst was first described as a suspicious adrenal mass, indicating the difficulty of diagnosis. The patient was completely asymptomatic, had no past medical records and took no medication. Physical examination was normal, as were urinary and blood laboratory tests. A subsequent MRI of the thorax showed a mass in the left adrenal compartment. The intensity of the mass was high on T2 and low on T1 weighed images, which excluded adrenal adenoma (Figure 2). Therefore, an adrenal adenoma was unlikely, yet not formally excluded. A tentative diagnosis of left diaphragmatic cyst was made.

**Figure 3:**
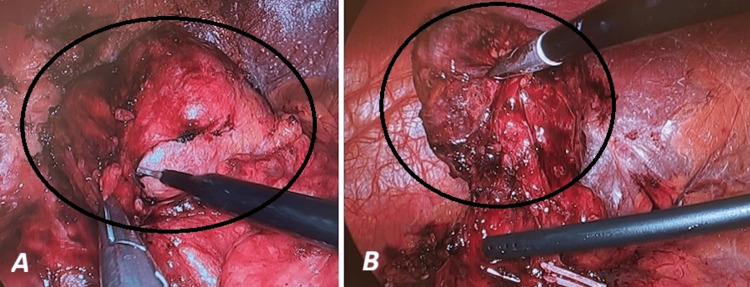
Exposure of the bronchogenic cyst (BC) A: Mobilisation of the splenic flexure of the colon and gentle retraction of the spleen were carried out to provide optimal access for BC (surrounded) isolation B: BC (surrounded) seems to be connected to the oesophagus

Despite advice to take a conservative approach, the patient elected to have the cyst removed. The patient consented to a minimally invasive laparoscopic approach. However, during surgery, once the left colon and spleen were mobilised, the mass appeared to be attached to the oesophagus instead of the diaphragm (Figure 3). 

**Figure 4:**
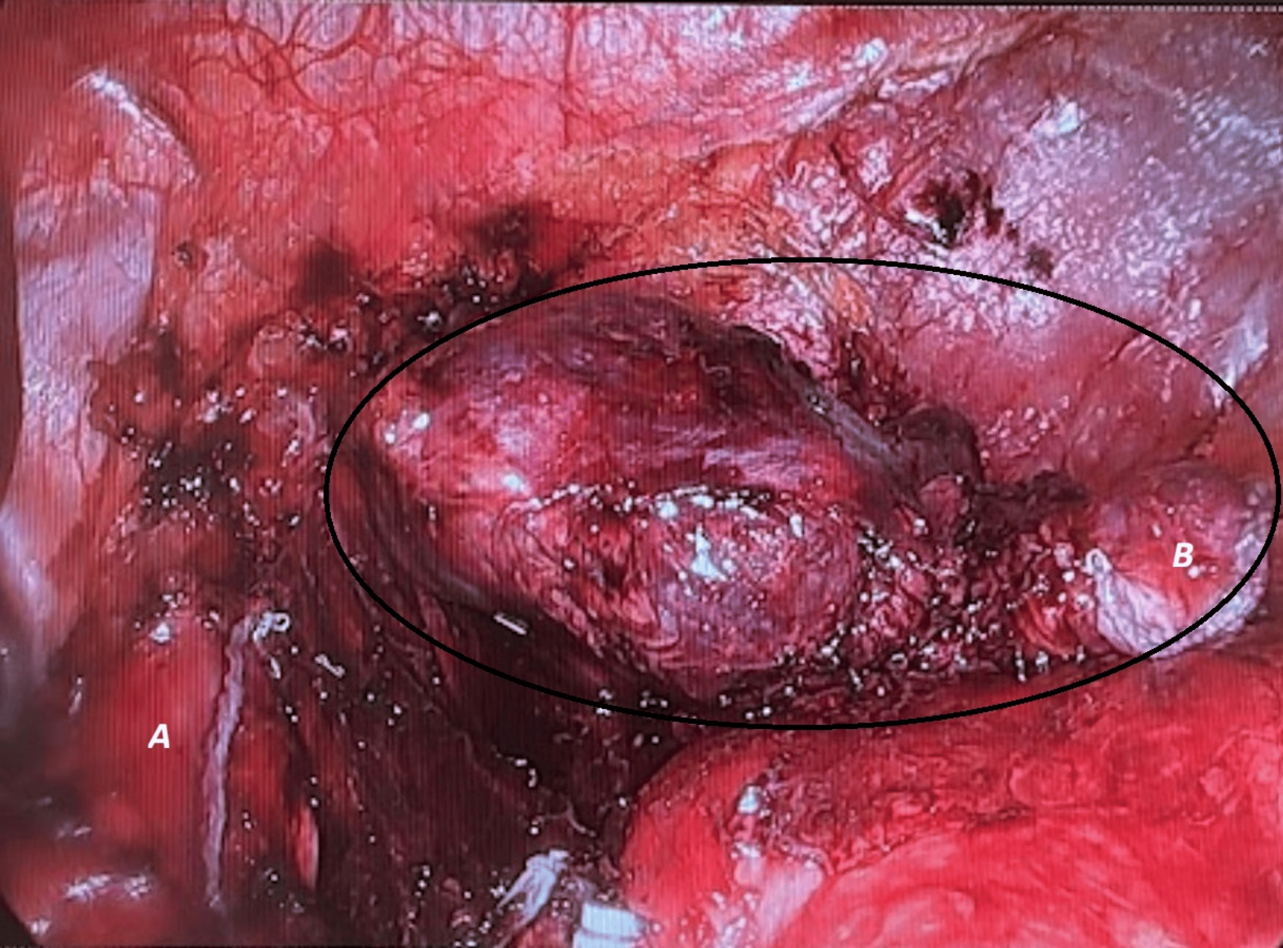
Dissection of the bronchogenic cyst (BC) The oesophagus (A) and cyst (B) seemed to be communicating. Complete detachment was achieved through stapling

Furthermore, careful examination uncovered an anatomical connection between the cyst and the oesophagus. We decided to detach it completely by stapling (Figure 4).

**Figure 5:**
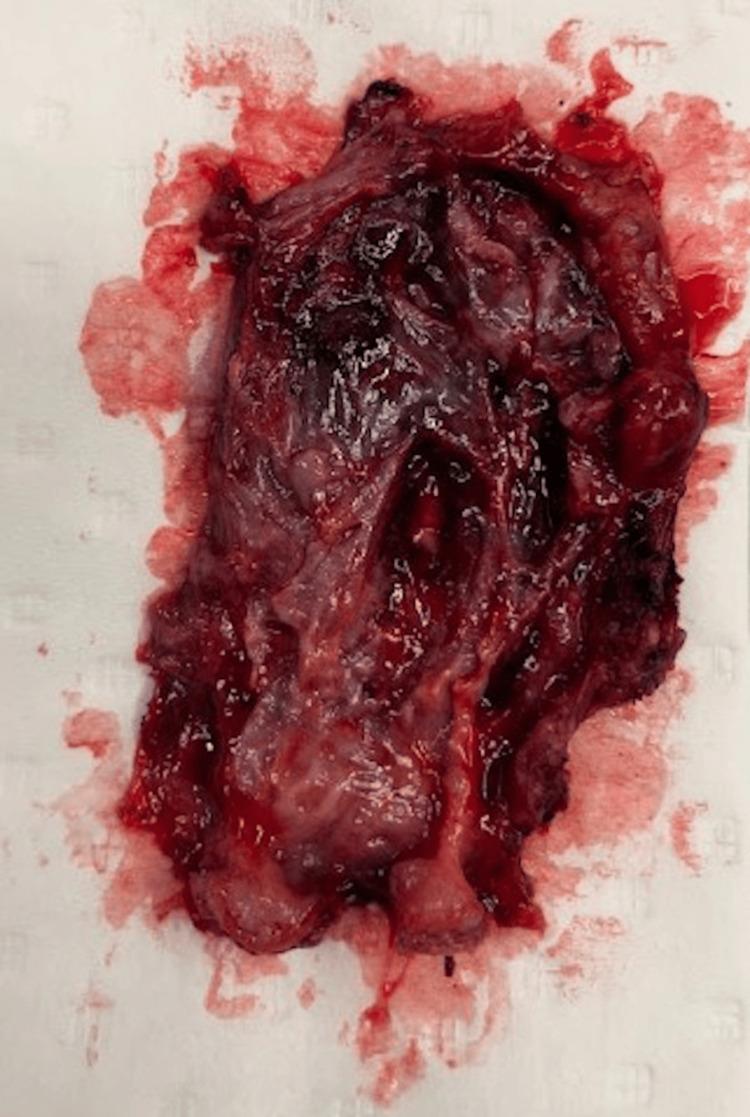
Anatomopathological specimen An ovoid cystic fragment measuring 6 × 3.5 × 0.5 cm, with necrotic areas

**Figure 6:**
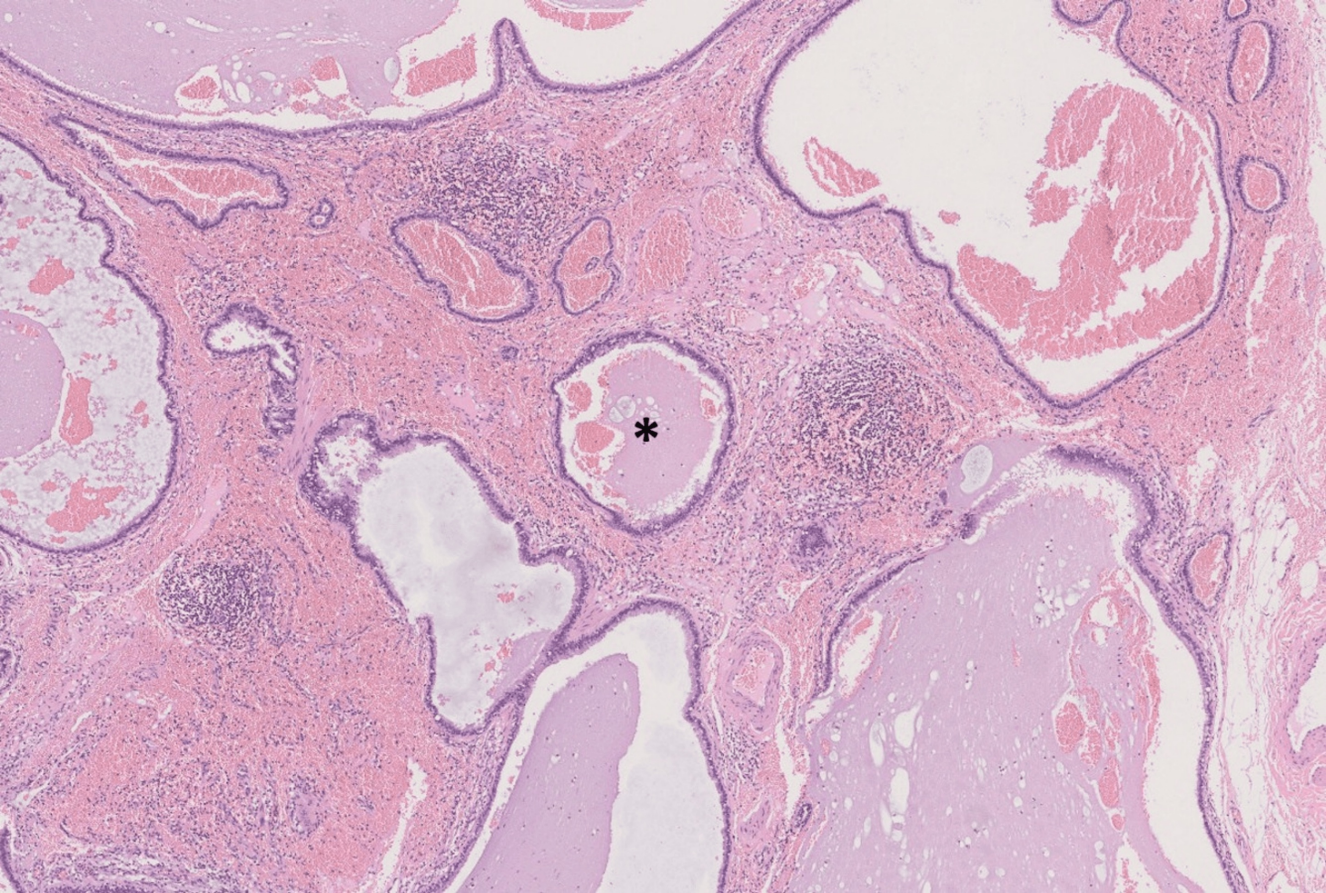
Anatomopathological slide Microscopic examination revealed a polycystic lesion lined by a ciliated pseudostratified columnar epithelium of respiratory type (*), associated with small congested blood vessels and a lymphoplasmacytic inflammatory infiltrate. A few cystic structures lined by a simple flattened epithelium without atypia were also identified, as well as normal cartilaginous tissue surrounding rare seromucinous glandular structures. The histological features, together with the anatomical location, were more suggestive of a bronchogenic cyst. No features suspicious for malignancy were identified.

Histological findings were compatible with a benign BC (Figures 5, 6). 

On postoperative day one, a radiographic swallow study showed no oesophageal leakage. Oral nutritional intake was increased adequately. Due to subcutaneous bleeding, tranexamic acid was administered, and low molecular weight heparin therapy was paused shortly. Subsequently, the postoperative course was uneventful.

## Discussion

A bronchogenic cyst (BC) is a generally benign, congenital malformation that arises from abnormal budding of the primitive foregut, usually occurring between the first and second months of gestation [2]. It affects about 1/55,000 people [2]. There are three types depending on their location: mediastinal, intrapulmonary, and ectopic [4]. Most are found in the mediastinum or lungs, but they can be found anywhere on the entire embryonic foregut pathway, including in the retroperitoneum, diaphragm, abdomen, sublingual cavities, and intracranial cavities. Approximately 10-15% of mediastinal tumors and 50-60% mediastinal cysts are BC, typically located in the middle mediastinum. Mediastinal BCs represent the majority of cases, typically making up roughly 50-70%. Intrapulmonary forms account for an estimated 20-30% of cases and tend to occur more frequently in males [2]. Gastric bronchogenic cysts, which constitute a rare ectopic variant, show a predominance in females, with an approximate female-to-male ratio of 21:14 [5].

A respiratory epithelium is characteristic of BC. The presence of cartilage, smooth muscle, and mucus-secreting glands in the histopathological specimen further reflects their airway origin. While the precise mechanism of abnormal foregut budding is not fully understood, it is believed that disruptions in key signaling pathways during critical stages of embryogenesis may lead to cyst formation [2]. BCs are most often discovered in the first years of life. When undiscovered, they can be asymptomatic for a long time due to their slow growth rate. The clinical presentation is highly variable, ranging from incidental findings to symptomatic masses causing pain, infection, or mass effect on adjacent structures [2]. They are then usually found incidentally on imaging, like in our case. As the clinical presentation is often nonspecific, imaging modalities like CT and MRI scans are key in distinguishing the BC from other (intra-abdominal) masses.

BC are typically circular or elliptical with well-defined margins [4-6]. The CT density in Hounsfield Units (HU) depends on its contents, being either water, mucus, or haemorrhagic fluid [6]. MRI planes can be useful in selected cases since it delineates anatomic relations better than CT scans [3]. When the diagnosis is still uncertain, an endoscopic ultrasound-guided fine-needle aspiration (EUS-FNA) can help to differentiate between benign and malignant lesions. In cases of intra-abdominal BC, EUS-FNA can assist in establishing a definitive diagnosis. This technique demonstrates a good sensitivity (93-95%) with a low complication rate (1-3%) [5]. Ulceration, infection, and hemorrhage are potential complications and can complicate subsequent surgical management because of potential adhesions [7]. The treatment of choice is a surgical complete resection [6-8]. Conservative treatment with watchful waiting and imaging follow-up is an acceptable alternative; however, the risk of secondary infection, ulceration, and in rare cases, malignant transformation should be taken into consideration [6].

Other differential diagnoses of abdominal BC include cervical cystic mass, cystic teratomas and germ cell tumors, esophageal duplication cysts, fungal diseases, hydatid cysts, thymic cysts, infected bullae, lymphangiomas, malignant lesions, neurogenic tumors, pericardial cysts, pulmonary abscess, pulmonary sequestrations, tuberculosis and vascular malformations [2-4].

## Conclusions

Bronchogenic cysts are rare congenital lesions that may occasionally present in ectopic locations, such as the abdomen, posing a significant diagnostic challenge. An accurate preoperative diagnosis is often difficult due to nonspecific imaging findings and the rarity of this entity. These observations highlight the need to consider bronchogenic cysts when investigating unusual intra-abdominal masses. We describe the diagnostic pathway that led to the final diagnosis to assist clinicians facing similar clinical scenarios in considering this rare entity. While the total excision of the mass remains debated, it is still considered the preferred treatment. Minimally invasive approaches, such as laparoscopy, offer excellent outcomes with reduced morbidity. We demonstrated that surgical removal by minimally invasive laparoscopy is safe and feasible, provided that a rigorous diagnostic work-up is conducted to rule out malignancy
